# Bacteriophages and related endolysins for reduction of microorganisms in the human body – a systematic review

**DOI:** 10.3205/dgkh000404

**Published:** 2022-01-17

**Authors:** Dilara Özal, Andreas Arndt, Marcus Thomé

**Affiliations:** 1 Kassel School of Medicine, University of Southampton, Southampton, UK; 2 B. Braun Medical AG, Sempach, Switzerland; 3 Department of Microbiology, Klinikum Kassel, Kassel, Germany

**Keywords:** antibiotics, antimicrobial resistance, bacteriophages, endolysins, infections, wounds, clinical trials, systematic review

## Abstract

**Background**: In recent years, resistance to antibiotics has become a global threat, and alternatives to antibiotics have become an area of research. The main alternative methods are briefly described in this review. However, the main focus is bacteriophage-related therapy.

Bacteriophages are viruses which, due to the production of the enzyme endolysin, are able to kill bacterial host cells. Bacteriophage therapies have a long tradition. Their potential to function as antibiotics lies in their bactericidal activity and specificity in killing bacteria without infecting or affecting eukaryotic cells.

**Objective**: To systematically review the outcomes of bacteriophage therapy in patients with bacterial infections.

**Methods**: The MEDLINE, EMBASE, Web of Science and CENTRAL databases were searched electronically using search terms referring to bacteriophages, endolysins and antimicrobial resistance. After the literature was screened for their titles and abstracts, full-text reviews considering inclusion/exclusion criteria were performed.

Data concerning patients with bacterial infections, treatment with either bacteriophages or its enzyme endolysin and their outcomes were extracted and analysed.

**Results**: Thirteen publications were identified that met all inclusion criteria. Data extraction shows that bacteriophages or endolysins have the potential to combat bacterial infections and significantly reduce inflammatory mediators. However, 3 out of 4 randomized controlled trials revealed that there was no significant difference between phage/endolysin treated patients and control group. Significant clinical improvements were seen in cohort and case studies. A few minor side effects were reported.

**Conclusions**: Although there are countries in which bacteriophages are prescribed as an alternative to established antibiotics, this valuable experience has yet to be examined sufficiently in clinical trials conducted to modern standards. Despite improvements in symptoms shown in the reviewed clinical trials, the infection and the bacteria themselves were rarely completely eradicated. Therefore, no definite answer can be given as to effectiveness, and further clinical trials are necessary.

## Zusammenfassung

**Hintergrund**: In den vergangenen Jahren hat die Resistenz gegen Antibiotika stetig zugenommen. Deshalb gewinnen synthetische und biotechnologisch basierte Strategien zur Therapie bakterieller Infektionen an Bedeutung. Das vorliegende Review konzentriert sich auf die Therapie mittels Bakteriophagen und ihrer Enzyme. Die langjährige Tradition der Phagen-Therapie beruht auf deren Spezifität, Bakterien abzutöten, eukaryotische Zellen jedoch weder zu infizieren noch zu beeinträchtigen. Eine wichtige Rolle spielen die von den Phagen produzierten Endolysine, welche in der Lage sind, die bakterielle Wirtszelle von innen zu zerstören.

**Ziel**: Systematische Überprüfung der Ergebnisse von Bakteriophagen bzw. Endolysin basierten Therapien bei Patienten mit bakteriellen Infektionen.

**Methoden**: Die Literaturrecherche konzentrierte sich auf die Datenbanken MEDLINE, EMBASE, Web of Science und CENTRAL mit Bezug auf Bakteriophagen, Endolysine und Antibiotikaresistenz. Nach Überprüfung der Literatur auf Titel und Abstracts, erfolgte eine Volltextüberprüfung anhand vorher definierter Einschluss-/Ausschlusskriterien.

Patientendaten mit bakteriellen Infektionen zur Behandlung mit Bakteriophagen oder Endolysin und deren Ergebnisse wurden extrahiert und analysiert.

**Ergebnisse**: Gesamthaft erfüllten 13 Publikationen die Einschlusskriterien. Die Datenextraktion zeigte, dass Bakteriophagen und Endolysine geeignet sind, bakterielle Infektionen zu bekämpfen sowie Entzündungsmediatoren signifikant zu reduzieren. Allerdings zeigten 3 von 4 randomisierten kontrollierten Studien, dass es keinen signifikanten Unterschied zwischen der mit Phagen bzw. mit Endolysin behandelten Patienten- und der Kontrollgruppe gab. In Kohorten- und Fallstudien wurde hingegen über signifikante klinische Verbesserungen berichtet. Es wurden nur wenige geringfügige Nebenwirkungen gemeldet.

**Schlussfolgerungen**: Obwohl es Länder gibt, in denen Bakteriophagen als Alternative zur etablierten Antibiotika-Therapie zum Einsatz kommen, mangelt es an klinischen Studien nach modernen Standards. Trotz des Rückgangs der Symptome bei diesen Therapien konnte nur selten eine vollständige Eliminierung der Infektion und der Bakterien selbst beobachtet werden. Um eine eindeutige Antwort auf die Wirksamkeit zu geben, sind weitere klinische Studien erforderlich.

## 1. Introduction

### Scope of the problem

Once harmful microorganisms invade the body’s tissues and multiply, they can cause damage and eventually lead to an infectious disease. Depending on the severity and type of infection, topical antiseptics or systemic antibiotic treatment are necessary. 

After the first antibiotic was discovered in 1928, the field of medicine underwent enormous changes. Before the antibiotic era, many infectious diseases were nearly impossible to treat [1]. Antibiotics are beneficial, as they can suppress the growth of bacteria, stopping them from reproducing and causing further damage to an organism. They are classified according to their mechanism of action [2]. 

Over time, some bacteria have developed resistance strategies against antibiotics, so they can withstand the medication. The molecular mechanisms of resistance are highly diverse and complex. Generally, resistance occurs due to the ability of bacteria to undergo structural and functional changes, which makes the antibiotic ineffective against bacteria [2], [3], [4].

In 2015, according to the European Center for Disease Prevention and Control (ECDC), there were more than 670,000 cases of diseases related with particularly dangerous, multi-drug resistant organisms (MRDO) in the European Union. In more than 33,000 cases, these were fatal [5]. The psychological and physical strain on patients and their relatives is enormous, not to mention the economic burden on public health systems made by multi-resistant pathogens [6]. Vancomycin-resistant enterococci (VRE), Methicillin-resistant Staphylococcus aureus (MRSA), third-generation cephalosporin-resistant Escherichia coli are among the most problematic MRDOs.

Antibiotic resistance thus numbers among the greatest threats to global health now and will continue to be so in the future, due to the increasing misuse, overuse and inadequate infection prevention and control. The World Health Organization (WHO) has introduced many strategies and policies to tackle antibiotic resistance and underline the fact that antibiotics should be used cautiously and with great awareness [7].

However, as revolutionary new antibiotics are not expected due to the increasing problem of antibiotic resistance, alternatives are a crucial area of research in the international medical community [8]. 

### Alternatives to antibiotics

A detailed overview of alternatives to antibiotics can be found both in Ghosh et al. [8], and others [9]. Two important alternatives are naturally occurring antimicrobials and synthetically designed strategies. Naturally occurring antimicrobial peptides (AMPs) are ancient evolutionary weapons stored in granules of phagocytic cells. They are found in all classes of life and function as the first line of defense against various pathogens [10], [11]. 

#### Synthetically designed strategies 

Chemists have developed strategies to modify or mimic natural AMPs [8], [12], [13]. One strategy involves attaching or covalently linking functional groups with antimicrobial activity, forming an antimicrobial polymer by maintaining the cationic and amphiphilic character of naturally occurring AMPs. Synthesized antimicrobial polymers show some evidence of having the potential for antimicrobial therapy and re-sensitizing drug-resistant bacteria [14]. 

The second approach is based on modification of the peptide backbone. These peptidomimetic approaches have oligomeric compounds enabling them to convert to secondary structures with antimicrobial activity [13], [15]. 

Lastly, small molecules are agents that mimic the properties of AMPs. Their modification relies on integrating facial amphiphilicity into small molecules via hydrogen bonding. In general, their pharmaceutical use is highly beneficial in terms of their synthesis, broad diversity and pharmacokinetics [12]. The effectiveness of small molecules in antimicrobial actions has been proven in a clinical trial [16]. 

Although synthetic mimics of antimicrobial peptides are one of the new generations of stable antimicrobial agents and have been used in different applications, its clinical use is still being studied and needs further clarification [13], [17].

#### Biotechnology-based approach

Bacteriophages (phages) are the most abundant and ubiquitous organism on earth, constituting an almost unlimited resource for researching the development of biomedical therapies. 

As phages are viruses, they need a bacterial host to survive. The virus benefits at the expense of the bacteria and eventually kills it, a process which will be described later. Developments in synthetic biology, such as high-throughput sequencing and genome editing have provided further understanding of bacteriophages [18]. Genetic modification promises to develop phages with unique properties for prophylactic and therapeutic applications. 

The “Traditional Homologous Recombination-Based Techniques” [18], involve the exchange of nucleotide sequences between homologous chromosomes that have similar or equivalent DNA regions [19]. This reaction will occur between at least two parental phages that carry the selective phenotypes. After recombination in their bacterial host cell, mutant phage progeny are screened and purified for further analysis [18]. A more specific modification of phage genomes is achieved by homologous recombination between plasmid and phage genomes. The chosen gene is first incorporated into the bacterial plasmid, resulting in recombinant phages with the desired genome [18], [20], [21].

Bacteriophage Recombineering of Electroporated DNA (BRED) is another frequently used engineering method that exploits a phage-encoded recombination system to enhance the frequency of recombination [18]. This is achieved by the co-electroporating phage DNA template and donor DNA, consisting of desired mutations, into bacterial cells, eventually leading to high levels of homologous recombination. After electroporation, plaques will contain both wild-type and mutant phages. The plates with plaques are screened by PCR. This method enables obtaining a high frequency of modified phages with only a small number of PCR screenings and no further selection is needed. BRED was initially used to modify mycobacteriophages, but it now also allows phages to target bacterial hosts such as *Escherichia* coli and *Salmonella enterica* [20].

#### Endolysins

Their natural function as enzyme-based antibiotics, also known as enzybiotics, has been proven recently through many different animal models [22], [23] and in food contamination [24]. However, its therapeutic use in humans is a new approach to research and will be explained in the discussion (below).

Generally, the peptidoglycan (PG) layer of bacteria is an essential structural component of the bacterial cell wall, necessary for protection, physical integrity and shape. Gram-negative bacteria consists of an outer membrane (OM) that lies above a thin PG layer. The OM is a semi-permeable membrane that hinders antimicrobial (including endolysins) access to the peptidoglycan layer [25]. Nevertheless, there is evidence that different types of endolysins are able to negotiate this barrier, but with a higher concentration than Gram-positive bacteria [26], [27]. 

Endolysins can have either a modular or globular structure. Those that infect Gram-negative bacteria are mostly small single-enzymatically active domain (EAD) globular proteins. In contrast, modular endolysins with multiple domains, including cell-wall binding domains (CBD), are unique to Gram-positive bacteria [24]. Modular structured endolysins can be engineered through the independent function of the catalytic domain (CD) and cell-wall binding domain (CBD), by fusing these two domains from various pathogens [25]. Artilysin is an endolysin with a specific outer-membrane (OM) permeabilizing peptide that degrades the PG layer of Gram-positive and Gram-negative bacteria, most notably *Pseudomonas aeruginosa* [28]. Several studies have revealed the broad-spectrum activity of chimeolysins against Gram-positive pathogens *Staphylococcus* and *Streptococcus* [29].

In summary, to overcome antibiotic resistance, researchers have focused on alternatives over the past several years. However, none of the alternative methods have definitively proven their effectiveness, and further research is necessary. Among the potential alternative therapies, phage therapy is one of the most promising approaches [9]. 

### History of bacteriophages

Bacteriophages were discovered in 1915 by Fredrick Twort. He observed “transparent” [30], areas on bacterial lawn, known as plaques. These plaques were the result of localized destruction of bacterial cells. However, Twort was unable to interpret his observations correctly. Félix Hubert d’Hérelle, independent of Twort’s findings, made a similar observation in 1917, noting that the plaques must be the result of an antagonistic microbe. Félix Hubert d’Hérelle provided evidence of his supposition when he isolated an “anti-Shiga microbe” from the stool of patients recovering from shigellosis. After he added his filtrate to a culture or an emulsion of Shiga bacilli, he was able to cause lysis of the bacilli [30], [31]. 

It is worth noting that there is some dispute as to whether d’Hérelle or Twort discovered bacteriophages first. Twort may have discovered bacteriophages in 1915, but his paper went unrecognized until 1921. Also, he was uncertain that he had found a bacterial virus. Although it is questionable that Félix Hubert d’Hérelle did not know of Twort’s discovery, d’Hérelle’s concluded that he had found a bacterial virus [31]. He was in contact with the Georgian Giorgia Eliava, who was also interested in the newly discovered bacteriophages. In 1923 d’Hérelle and Eliava opened the Eliava Institute for Bacteriophages, Microbiology and Virology in Tbilisi.

Phage therapy was already being used in the USSR during the Soviet-Finnish War and during World War II. These historical data are not considered in this review but are given elsewhere [32], [33].

The declining enthusiasm for bacteriophages especially in the Western World started with the discovery of penicillin in 1928. Antibiotics were the more convenient solution, as they were easier to produce in large quantities, chemically stable and uniformly active on many bacteria [34].

### Bacteriophage mechanism of action

**Figure 1:**
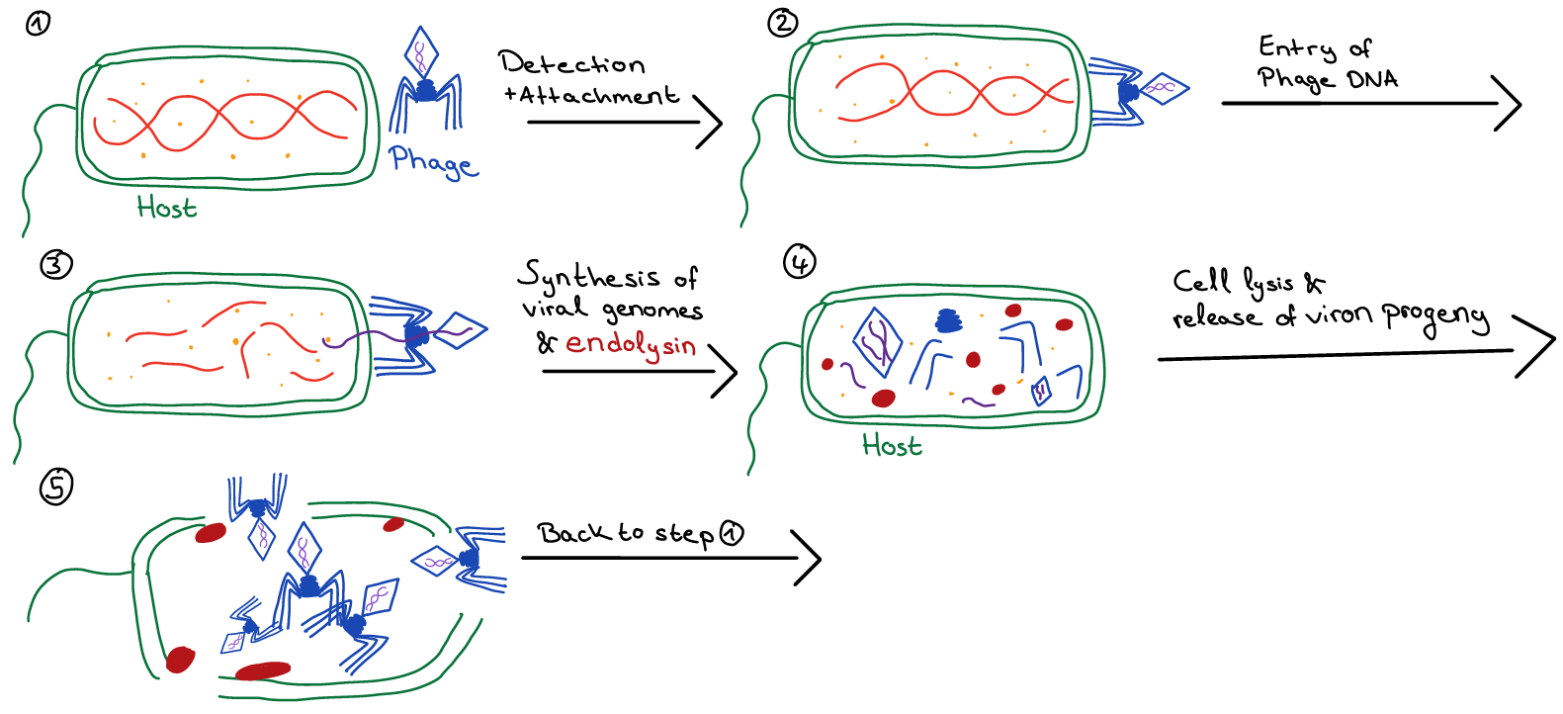
Bacteriophage mechanism of action

As shown in Figure 1 (Fig. 1), the phage detects its host bacterium from the surface structure (1). It attaches to the surface and injects its genetic material into the bacterium (2). At this stage, the bacterium can live and reproduce normally, and phages use bacterial enzymes to replicate and produce multiple copies of itself within the bacterial host cell. At the end of the lytic cycle, the bacteriophage releases endolysins that allows dispersion of virion progeny (3). Starting with a single phage, several dozen similar phages are created that search for other bacteria to infect (5) [34], [35]. 

Endolysins, also known as phage-encoded-peptidoglycan hydrolases (PGH), enzymatically degrade the peptidoglycan layer of the bacteria. However, they do not have any signal sequences that allow direct access to peptidoglycan. Thus, a second protein, holin, which is produced during the replication cycle, is needed. Holin forms holes in the bacterial cell wall to allow endolysin to reach the peptidoglycan layer and degrade it [35].

There are two general classes of bacteriophage lytic enzymes: endolysin and virion-associated peptidoglycan hydrolases (VAPGH). Although both display antimicrobial activity, they act at different times during the lytic cycle. VAPGHs are necessary for generating a hole in the bacterial cell wall at the start of the infection cycle, making the injection of the viral genetic material possible via the phage’s tube tail. Endolysin, however, mediates lysis at the end of the lytic cycle, as previously described [36].

Depending on the type of phage, there are two ways to achieve replication [34]. One is the lytic cycle, in which bacterial cells lyse immediately after replication, making it more useful in phage therapy.

Replication via lysogen does not lead to immediate lysing of the host cell, as the viral genome remains endogenously dormant, unless the host weakens and the process of lysing can be initiated [30], [35]. 

### Phages as antibacterial drugs

The first step of phage therapy involves phage isolation and choice. After the pathogenic bacteria have been detected, the phages are mostly chosen in the form of a cocktail that has a wide spectrum of activity. The isolated phages are then tested against the bacteria that cause the infection. Purification is finally needed by clarifying lysed culture, centrifuging, filtering to remove any extraneous matter [34].

Plaque forming units (PFU) are a measure of the number of infectious virus particles, and is determined by plaque forming assay [37]. 

Bacteriophages are ultimately replicated by their host. As soon as the host is no longer available, the phage can no longer multiply and phages will die off. The fact that viruses are specific to their host means that the therapy would only attack the pathogen, unlike antibiotics [34]. 

All of these are properties that make bacteriophages extremely useful as a biological antibiotic.

## 2. Methods

This report was based on the Reporting Items for Systematic Reviews and Meta-Analyses (PRISMA) guidelines (see Tab. 1 in Attachment 1). An initial scoping search of the databases DelphiS (Health-Science-Library University of Southampton) and Prospero was run to avoid any overlaps among published studies. 

### Eligibility criteria

To define eligibility criteria, the PICO (ST) model for medical research (see Tab. 1 in Attachment 1) was used.

**Population/problem**

- Individuals with bacterial infections
- Individuals resistant to antibiotics

**Intervention**

- Individuals who received bacteriophage treatment with a certain type of enzyme, a certain dose and for a certain length of time

Comparison

- The interventions stated were compared with patients who received antibiotics, other supplementary treatments or placebo

**Outcome**

- Wound healing
- Reduction of infection
- Any positive or adverse outcome

**Study design**

- Case-control studies
- Case studies
- Cohort studies
- Experimental trials
- Clinical trials

**Setting**

- Healthcare setting

**Timing**

- No date restrictions to ensure all relevant studies were identified

#### Inclusion criteria

Patients with bacterial infections that were treated with bacteriophages or endolysins in a healthcare setting. Control groups that received antibiotics, other supplementary treatment or no treatment at all were included. 

#### Exclusion criteria

Healthy patients or those who had different types of infections were excluded. Studies based on different alternative therapies were not included. Patients treated with additional antibiotics were excluded. Animal and in-vitro studies were excluded. 

### Search strategy and databases

Four medical databases were searched in October 2019: Medline (Ovid), EMBASE (Ovid), CENTRAL (Cochrane Library’s Central Register of Controlled Trials) and Web of Science (see Tab. 6 in Attachment 1). The search comprised four search strings: enzyme therapy, antibiotics, reduction of infection and human. These terms were combined with free-text words by the Boolean operators in order to find all the relevant papers. 

The results were limited to human studies, German and English language only.

Hand searching and citation chaining for additional relevant literature was conducted. Furthermore, a search of grey literature was conducted. 

Finally, corresponding authors and clinics were contacted for further information about studies and two papers [38], [39] due to missing data. From these, results of one study could be obtained from a different paper [40].

### Study selection

All the papers were exported to the reference manager Endnote Online, and duplicates were removed. Study selection involved two steps:

- Titles and abstracts were screened for eligibility, and irrelevant papers were rejected
- Full texts of potentially eligible studies were assessed according to the inclusion and exclusion criteria. Reasons for exclusion were noted (see Tab. 2 in Attachment 1)

### Data extraction

An electronic data table for data extraction was designed and piloted. Extracted data included:

- Study characteristics (title, author, year, study design, country, intervention, control, outcomes, follow-up period)
- Participant characteristics (number of patients and controls, loss to follow-up and reason, gender, mean age, type and duration of infection, pathogen, mean disease duration)
- Study results

### Data synthesis

A narrative data synthesis was conducted. 

### Risk of bias assessment

Due to the different study types included in this review, more than one critical appraisal tool was necessary. The Critical Appraisal Skills Programme (CASP) quality assessment tool offers comparable checklists for different study designs [41], [42], making it an appropriate tool. A slightly modified CASP cohort study checklist was used for two prospective studies and one retrospective study, while the CASP checklist for randomized controlled trials was used for 4 RCTs to assess the quality of each study and ensure detection of relevant flaws.

For case studies, Joanna Brigs Institute (JBI) Critical Appraisal Checklists were used (see Appendix 7.4 in [43]). Quality assessment was conducted after data extraction to minimize reporting bias.

## 4. Results

**Figure 2:**
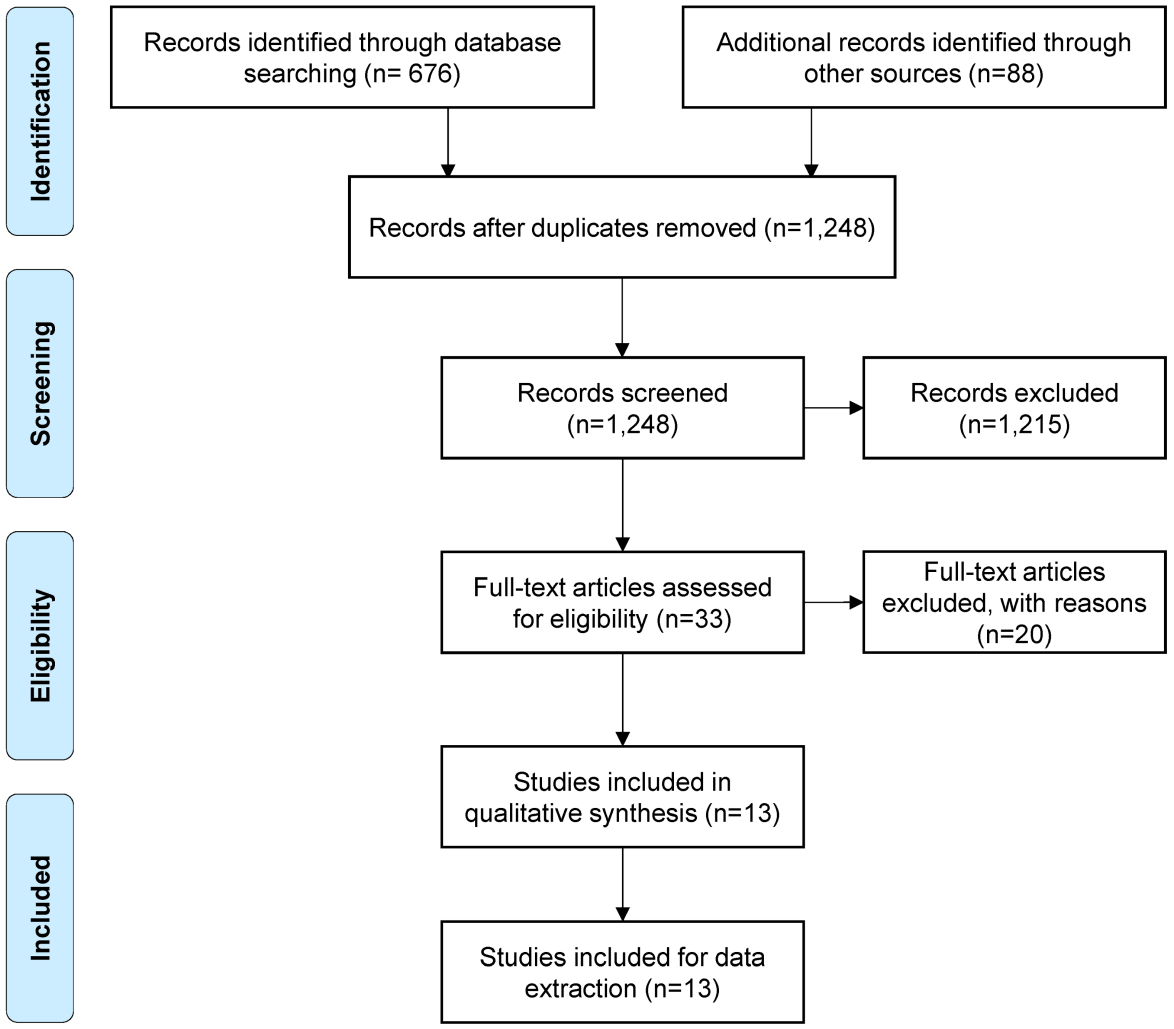
Prisma flow diagram [57]

The systematic search yielded 1,676 papers. After removing duplicates and the two previously described screening strategies were employed, 13 papers were assessed for eligibility (Figure 2 (Fig. 2)). 

### Study characteristics

#### Patients and controls

Participants were recruited from the USA [44], [45], [46], [47], Poland [48], the UK [49], [50], Belgium [51], India [52], [53], and the Netherlands [39], [54]. One study recruited patients in Belgium and France [55]. From these, five studies had control groups that were treated with corticosteroid cream [39], antibiotics [55], saline [44], [49] or antibiotics in addition to bacteriophages [48].

In the RCTs, the numbers of included patients and controls ranged from 12 [49], [55] to 50 [39]. The number of patients who received bacteriophage therapy in cohort studies varied between 20 [52] and 48 [53]. The age of the patients ranged from 15 [50], to 92 years [45]. 

Disease duration varied from 6 weeks [53] to 58 years [49], and the duration of treatment varied between 7 days [55], and 8 months [50].Two studies [45], [46] treated diabetic food ulcer (DFU), one study treated cystic fibrosis [50] and burns, [55] two studies treated septicemia [48], [51], and the rest treated different types of chronic diseases [39], [44], [47], [48], [49], [50], [51], [52], [53], [54]. 

#### Measures

All 13 studies used different types of bacteriophages and different methods to treat the infections. One RCT and one case study used endolysins as treatment [39], [54]. Some of the studies used commercially available bacteriophages [39],[45], [46]. 

Studies used intravenous [47], [50], [51], oral [48], otic [56], percutaneous [47], and subcutaneous [46], application routes, all other 7 studies used topical administration.

#### Outcome

All studies focused on the safety and efficacy of bacteriophages (Table 1 (Tab. 1)). Three studies reported adverse events [44], [49], [55]. 

#### Study design

The 13 studies were classified into the following: 4 double-blinded randomized controlled trials [39], [44], [49], [55], 2 prospective cohort studies [52], [53], and one retrospective cohort study [48]. The other studies were either case series [45], or case reports [46], [47], [50], [51], [54].

### Results of studies

#### Bacteriophage-related treatment of chronic diseases and ulcers

Treatment of chronic wound infections is very complex, with a significant burden being placed on patients and medical systems. If physiological processes are not able to heal wounds, they remain in the inflammatory phase and are thus diagnosed as chronic wounds [57]. 

In a case study, patients with *S. aureus*-related skin disorders were successfully treated with Staphefekt™ SA.100. Staphefekt™ SA 100 is an engineered endolysin that is an active compound of cetomacrogol-based cream, and available in Europe as an over-the-counter treatment [54].

Different result were shown in a study protocol for a RCT by Totté et al [39].

In this clinical trial the difference in the need for topical corticosteroid (TCS) co-therapy between the Staphefekt and the placebo group was assessed, measuring the number of days per week of corticosteroid cream (triamcinolone) use. Data demonstrate that patients in the endolysin group used TCS for 1,889 days (45.0%), while in the control group (triamcinolone only). it was used for 1,566 days (37.3%). Both studies showed no significance in terms of *S. aureus* reduction and no therapy-related adverse effects were reported. 

According to Rhoads et al. [44], there is no significant difference in healing frequency or in healing rates after 12 weeks of treatment between control and phage-treated patients. 

However, successful treatment of DFU in a patient with osteomyelitis and methicillin-sensitive *Staphylococcus aureus* (MSSA) was achieved by subcutaneously injecting phage-Sb1 into soft tissue once a week for seven weeks [46]. Similar results were observed in a different study [45].

In two prospective cohort studies, from the same medical institute in India, there was a significant clinical improvement of chronic wounds [53], [52]. 39/48 [53], of patients and 7/20 [52] were completely cured. 

A randomised study on chronic otitis media patients [49], reported significant improvements of patients who randomly received a single application of bacteriophages (105 PFU/ml). The measure was based on a Visual Analogue Scale (VAS). Further findings involve the significant reduction of *P. aeruginosa* counts on days 21 and 42. It is important to note that after resolution, the infection clearance of bacteriophages was observed and clinical scores rose again.

Lastly, improvements in lung and liver function were observed with intravenous bacteriophage application [50]. 

#### Phage therapy of infected burns and sepsis

A multi-center trial [55] investigated the efficacy and tolerability of a cocktail of lytic anti-pseudomonas aeruginosa bacteriophages, compared with standard care for patients with burns. The results show that the median time of sustained semiquantitative reduction of two or more quadrants was significantly longer in phage-treated patients. Clinical improvements such as pus and the closing of wounds were less frequent in the standard of care group [55].

A case report confirmed the effectiveness of intravenous application of BFC-1 bacteriophages against *P. aeruginosa* in a septic patient. Blood cultures became negative and inflammatory mediators dropped rapidly. Topical BFC1 therapy did not show any positive results [51]. 

### Quality appraisal

The quality of the studies was assessed using appropriate quality assessment tools (see Tab. 3, Tab. 4, and Tab. 5 in Attachment 1). Due to the different study types, different checklists were used. A study that fulfilled fewer checkpoints indicates a higher vulnerability. If a study met half or more of the criteria on the checklists, it was considered as an acceptable study. Information was taken from published studies and supplementary appendices.

For RCT, the Consolidated Standard of Reporting Trials (CONSORT) and Critical Appraisal Skills Program (CASP) checklist were used to assess the quality of each study, as well as to detect any relevant flaws. Remarkably, all of the RCTs were double-blinded, except for one study [55] in which it was not possible due to the different appearances of the two treatments. Homogeneity among treatment and control groups was only partially achieved. In one study, the mean age group in the treatment group was much higher than in the control group, but they were less extensively burned [55]. Another study lacked information about the groups [49]. 

The other two RCTs had similar treatment and control groups [44], [49]. Only two studies avoided possible selection bias by providing information about the method of the allocation sequence [39], [55]. Two studies did not clearly state how randomization was achieved [44], [49]. 

The cohort studies were evaluated with the CASP [41] checklist. None of the 3 studies accounted for confounding factors in the statistical analysis, resulting in distortion of the apparent effect, thus risk of reporting bias. Notably, most of the participants within a cohort study received the same treating agent and therefore lacked a control arm. All except one study looked at patients who had been treated either with bacteriophages alone or bacteriophages in addition to antibiotics [48]. However, detection bias may have been introduced based on the retrospective linkage. Also, this study did not include all patients in the results; thus, the published results do not properly reflect the results of the study. Due to the fact that the cohort studies were open-label, a selection bias may have occurred. 

Case studies were analyzed with the Joanna Briggs Critical Appraisal Checklist [43]. Four [46], [50], [51], [54] case studies had clear presentation of the patient’s history. The presence of no adverse events was reported in some patients and not stated at all in other cases [46]. Lastly, there were four author teams [39], [52], [53], [54] who recruited their patients from the same clinic, which increases the risk of overlaps in participants.

## 5. Discussion

This manuscript used systematic review methodology to investigate the outcome of bacteriophage-related therapy in humans.

### Summary of evidence

The safety of bacteriophage-related therapy has been confirmed in several clinical trials. The studies used different strategies and routes of application, with topical administration being the most common. Nevertheless, studies with different types of drug administration were still successful. Successful therapy was mostly seen in studies that used a dosage of >106 PFU/ml phage preparation. It must be mentioned that not all studies stated the concentration. Remarkably, one study used a dose of 105 PFU/ml, but the results were still clinically successful [49]. 

Successful treatment also depends on the type of bacteria, and most successful results were seen in *P. aeruginosa* and *S. aureus* infections. 

In case studies. the patient had suffered for at least 3 months from infections, with various forms of exacerbation. Phage-related therapy was, therefore, often their last treatment option. 

Eight studies showed local improvements, e.g., wound healing, reduction of redness and pustules; systemic improvements, such as the reduction of bacterial count, fever and CRP level, were observed in 5 studies. Recurrence of the infection was documented in two studies [49], [54]. Lastly, mild to moderate adverse effects were reported in 2 studies [44], [49]. 

### Challenges of phage-related therapies

Although there has been successful treatment with this alternative therapy, there are many different aspects of this therapy that should be considered carefully. 

#### Neutralization of treatment

Bacteriophages show remarkable action against anti-inflammatory mediators. In some of the studies, there was a significant reduction of C reactive protein (CRP) and leukocyte counts [48], [51], [53]. 

One study revealed that phages administered orally and/or locally 2–3 times daily, resulted in a significant decrease in mean CRP and mean white blood cell count (WBC) [58]. 

It is worth noting that the production of antibodies has the potential to prevent the efficacy of phage therapy [59], but at the same time there is evidence that phage therapy is mostly completed before the natural immune response begins [60]. This was observed in the case report where antibody responses started after one month of treatment [50]. Generally, neutralisation of phage-related therapy was not observed in the included studies.

A different study concluded that the route of phage administration plays a significant role in anti-phage activity, as assessed by sera in patients. Participants of the study who received the phages orally, showed the highest anti-phage activity [61]. 

Interpretations of the impact of bacteriophages on human immunity and their antibacterial efficacy have been quite contradictory and unclear, because anti-phage antibody production does not necessarily affect the outcome of treatment.

Other factors, such as duration of treatment and phage dosage, should be considered in further studies, in order to provide better understanding of immune responses against phage therapy.

#### Pharmacological limitations 

The greatest challenge in phage therapy is ensuring quality and safety at every stage of production, as phages are a biological entity and biotechnological production is a far more complicated than chemical synthesis.

For example, the “PhagoBurn” [55] project was terminated prematurely due to the ineffectiveness of the phage preparations. The problem was that the liquid preparations of the phages were unstable, and the phage concentration was therefore far lower than expected [55]. A similar phenomenon had occurred in a clinical study funded by Nestlé, to test a drug product against diarrhea in children from Bangladesh. The study was also prematurely terminated because the tested preparation, consisting of the Escherichia-coli Phage T4, showed no better results than the standard therapy [62]. Formulations of bacteriophages as therapeutic application thus requires careful chemical and physical techniques for encapsulation. The most common methods for stabilizing and encapsulating phages are spray drying, freeze drying and extrusion dripping methods [56].

In addition to instability, research into phage therapy examines several routes of administration. For example, one study exposed phages to ultrasound, and it is questionable whether this may have affected the viability of phages and the non-significant results of healing [44].

It is technically not possible to produce two batches of a phage cocktail that are exactly the same. Replication inevitably creates small mutations that are randomly distributed across the genome. Furthermore, it is important that the phages not be lysogenic, as via horizontal gene transfer, pathogenic properties of their host can be transmitted to other bacteria. In addition, the preparations must remain below predefined limit values for contaminants, such as bacterial endotoxin [6]. 

#### Insufficient guidelines

Such a dynamic product places special demands on clinical testing, as well as potential approval, and these are simply not addressed by the current regulations. In Europe, a product needs to have the status of a medication in order to be allowed for clinical use. 

The European guidelines for clinical trials and the standards of the International Conference on Harmonization, as well as good manufacturing practice (GMP), are decisive for the approval of biological or biotechnological products. The German authority responsible for the approval of medicinal products, the Federal Institute for Drugs and Medical Devices (BfArM), admits that these are insufficient for bacteriophages. On an international level, however, it was already possible to agree on some critical quality attributes (CQA) that must be achieved, and thus at least enable GMP-compliant phage production.

Therefore, the clinical use of bacteriophages has yet to be legally confirmed for obtaining comprehensive information about phage therapy [6]. 

#### New endolysin trials

Besides the endolysin Staphefekt SA.100 [39], [54], there are some new endolysins that have been tested, mainly on healthy volunteers, in order to evaluate their pharmacokinetics, pharmacodynamics and tolerance. 

For example, SAL 200^®^ was administered intravenously in a single dose to 34 healthy volunteers. The results showed a high tolerance against staphylococcal endolysin. Phase II of this study has recently started to explore the efficacy of single intravenous doses of SAL200^®^, in addition to the conventional standard treatment [63]. 

Another ongoing trial is for Medolysin^®^ as a wound spray, which forms a protective film and reduces bacterial load on the wound [64].

The ContraFect company assessed safety and tolerability of endolysin Exebacase (CF-301). Out of 20 healthy volunteers, no adverse clinical effects were observed [65]. In this randomized safety test, patients received a single intravenous dose of CF-301 or a placebo for 2 h. Phase II of this study, for the treatment of *S. aureus* bacteremia, including endocarditis, was recently completed. 

The clinically significant improvements among patients treated with Exebacase in addition to standard of care (SOC) antibacterial therapy, when compared to SOC alone, were announced 2016, at the European Congress of Clinical Micobiology & Infectious Diseases (ECCMID). 

The efficacy of endolysin therapy has been shown in several animal studies [66], [67], [68], but research on effectiveness, resistance or allergenicity in human trials is still ongoing. One unique property of endolysins is their specificity. Like phages, they will not harm the microflora. Furthermore, they rapidly cause bacterial lysis, while antibiotics depend on the inhibition of a metabolic step within the bacterial cell, which is a slower process.

Lastly, the problem of narrow spectrum activity can be solved, for example, by combining different endolysin domains that result in a broader lytic spectrum [27]. 

### Limitations and strengths of included studies

Because 6 studies had similar author groups, there is a high risk that the same patients were involved in more than one study. This increases the risk of selection bias, and the results may not be representative for the particular disease. 

However, all RCTs were of high methodological quality. The method of randomization and concealed allocation was well described in all four studies.

Only one of the studies [39] performed a power calculation to obtain an appropriate patient sample size. This might be because only a small number of patients agreed to be treated with a not fully approved drug. A low power makes a study more vulnerable to type-II error. Therefore, the studies that found no differences between patients and controls might thus be subject to type-II error. By increasing the sample size, which is difficult with a non-approved drug, or by reducing the variability in a patient sample, the power of the study can be increased.

Some of the studies were funded by companies, and in some studies, commercial phages were used. Only a few studies stated that the funders were not involved in data interpretation, and thus funding bias should be considered. 

A further limitation is the unspecific description of the applied phages, in terms of their type and concentration. 

### Limitations and strengths of the review process

Ideally, the searches, screening stages and critical appraisal of a systematic review should be conducted by two researchers. 

For reasons of time and budget constraints, this was not feasible in this review. This limitation was addressed through close monitoring and discussion with the supervisor. To minimize the risk of bias, all decisions were checked twice, and transparently recorded and reported. A second limitation might be that patients who were treated with additional antibiotics or the drug Phagobioderm, a biodegradable polymer impregnated with ciprofloxacin and bacteriophages, were excluded. Synergistic effects of antibiotics and phage-related therapy thus were not considered. In one study, patients were treated before and during the trial with additional triamcinolone, which might have prevented a possible benefit of endolysin treatment. 

To ensure all relevant studies were identified, there was no date restriction. However, some older studies were excluded, due to a lack of accuracy and language barriers.

The actual therapy with Phages are still more common in the states of the former Soviet Union, in particular in Georgia. Thus, studies that were not available in English or with an English abstract only, were excluded. In this way, further important findings might have been excluded.

Nevertheless, we decided to prioritize more specific studies over the quantity of less specific studies. Therefore, a more detailed analysis and appraisal of each study was possible.

Although 13 included papers in this review is not a large number, piloting of the search strategy and supplementation of electronic searches by hand and reference searching provides confidence that all the relevant research was included in this systematic review of all available evidence. 

### Recommendations for future research

Although there are countries in which bacteriophages are prescribed as an alternative to established antibiotics, this valuable experience is not backed by clinical trials conducted to modern standards.

Future research should address the methodological and conceptual limitations of the currently published findings. It should aim to optimize manufacturing processes and study designs, in order to ensure a representative assessment of phage-related therapy as a novel treatment option. Larger sample sizes, without losing the homogeneity of patients, could be achieved through multi-center studies.

Combining bacteriophages with antibiotics to maximize effectiveness and minimize resistance should be considered. 

## 6. Conclusions

Uncertainty in the available clinical data means there is insufficient evidence draw conclusions about the outcomes of bacteriophage-related therapy. 

We have identified a range of different disorders in which these therapies were effective. Despite improvements in symptoms with these alternative therapies, total eradication of the infection and the bacteria themselves was rarely observed. This raises the question of whether total eradication is necessary for clinical improvements. 

Although this review included important findings, no definite answer can be given about phage-therapy effectiveness, and larger clinical trials are necessary.

## Notes

### Competing interests

The authors declare that they have no competing interests.

## Supplementary Material

Full details of study (Tab. 1–6)

## Figures and Tables

**Table 1:**
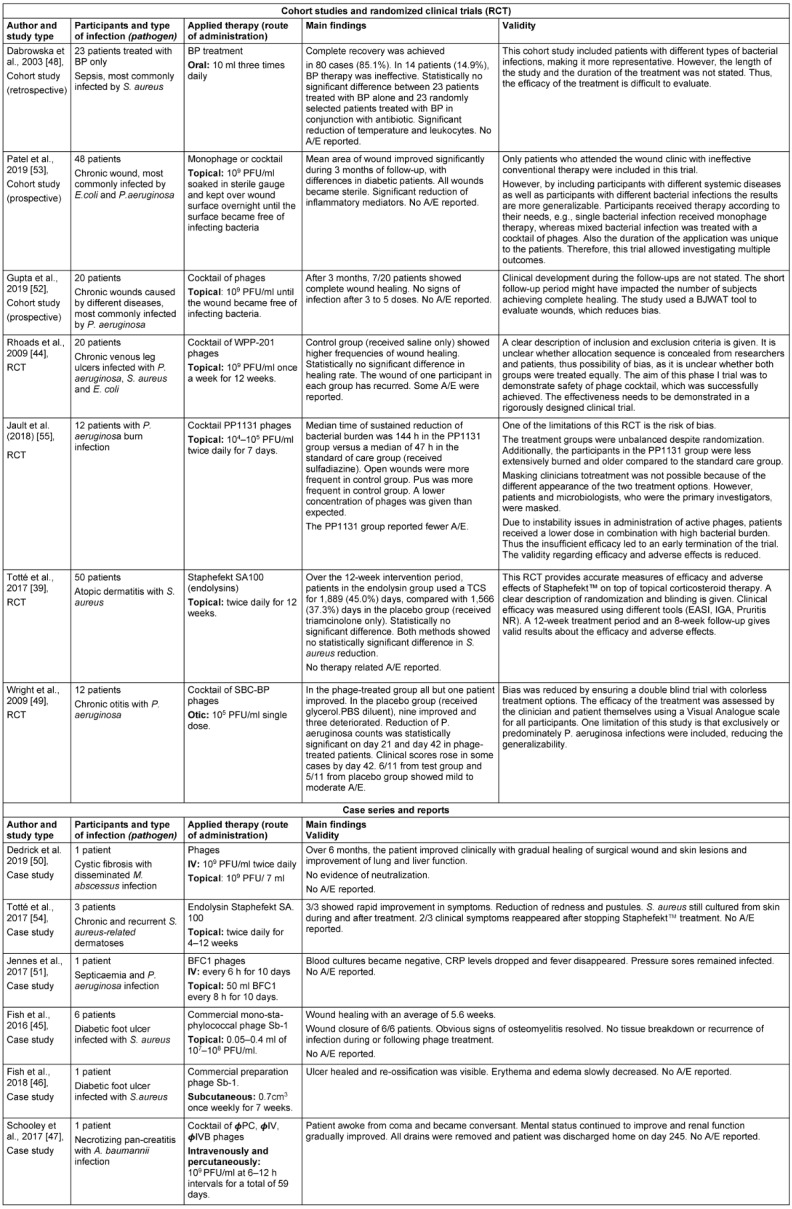
Data extraction summarized
