# Supplementary material for: Bacteriophages and related endolysins for reduction of microorganisms in the human body – a systematic review
Source: GMS Hyg Infect Control. 2022 Jan 17;17:Doc01. doi: 10.3205/dgkh000404 (PMC8780682; doi:10.3205/dgkh000404)
Supplement: Full details of study (Tab. 1–6) [file HIC-17-01-s-001.pdf]

Attachment 1

Tab. 1: PRISMA 2009 Checklist
Rahim K, Saleha S, Zhu X, Huo L, Basit A, Franco OL. Bacterial Contribution in Chronicity of Wounds. Microb Ecol. 2017 Apr;73(3):710-21. DOI: 10.1007/s00248-016-0867-9

Section/topic	#	Checklist item	Reported on page #
**TITLE**			
Title	1	Identify the report as a systematic review, meta-analysis, or both.	1
**ABSTRACT**			
Structured summary	2	Provide a structured summary including, as applicable: background; objectives; data sources; study eligibility criteria, participants, and interventions; study appraisal and synthesis methods; results; limitations; conclusions and implications of key findings; systematic review registration number.	4
**INTRODUCTION**			
Rationale	3	Describe the rationale for the review in the context of what is already known.	9-17
Objectives	4	Provide an explicit statement of questions being addressed with reference to participants, interventions, comparisons, outcomes, and study design (PICOS).	18
**METHODS**			
Protocol and registration	5	Indicate if a review protocol exists, if and where it can be accessed (e.g., Web address), and, if available, provide registration information including registration number.	n.a.
Eligibility criteria	6	Specify study characteristics (e.g., PICOS, length of follow-up) and report characteristics (e.g., years considered, language, publication status) used as criteria for eligibility, giving rationale.	18-19
Information sources	7	Describe all information sources (e.g., databases with dates of coverage, contact with study authors to identify additional studies) in the search and date last searched.	20
Search	8	Present full electronic search strategy for at least one database, including any limits used, such that it could be repeated.	52
Study selection	9	State the process for selecting studies (i.e., screening, eligibility, included in systematic review, and, if applicable, included in the meta-analysis).	19-20
Data collection process	10	Describe method of data extraction from reports (e.g., piloted forms, independently, in duplicate) and any processes for obtaining and confirming data from investigators.	20
Data items	11	List and define all variables for which data were sought (e.g., PICOS, funding sources) and any assumptions and simplifications made.	18-19
Risk of bias in individual studies	12	Describe methods used for assessing risk of bias of individual studies (including specification of whether this was done at the study or outcome level), and how this information is to be used in any data synthesis.	20
Summary measures	13	State the principal summary measures (e.g., risk ratio, difference in means).	n.a.
Synthesis of results	14	Describe the methods of handling data and combining results of studies, if done, including measures of consistency (e.g., I^2) for each meta-analysis.	21

Attachment 1

Section/topic	#	Checklist item	Reported on page #
Risk of bias across studies	15	Specify any assessment of risk of bias that may affect the cumulative evidence (e.g., publication bias, selective reporting within studies).	20; 29
Additional analyses	16	Describe methods of additional analyses (e.g., sensitivity or subgroup analyses, meta-regression), if done, indicating which were pre-specified.	n.a.
**RESULTS**			
Study selection	17	Give numbers of studies screened, assessed for eligibility, and included in the review, with reasons for exclusions at each stage, ideally with a flow diagram.	21; 47
Study characteristics	18	For each study, present characteristics for which data were extracted (e.g., study size, PICOS, follow-up period) and provide the citations.	29
Risk of bias within studies	19	Present data on risk of bias of each study and, if available, any outcome level assessment (see item 12).	47
Results of individual studies	20	For all outcomes considered (benefits or harms), present, for each study: (a) simple summary data for each intervention group (b) effect estimates and confidence intervals, ideally with a forest plot.	22-26
Synthesis of results	21	Present results of each meta-analysis done, including confidence intervals and measures of consistency.	n.a.
Risk of bias across studies	22	Present results of any assessment of risk of bias across studies (see Item 15).	20; 29
Additional analysis	23	Give results of additional analyses, if done (e.g., sensitivity or subgroup analyses, meta-regression [see Item 16],).	n.a.
**DISCUSSION**			
Summary of evidence	24	Summarize the main findings including the strength of evidence for each main outcome; consider their relevance to key groups (e.g., healthcare providers, users, and policy makers).	31
Limitations	25	Discuss limitations at study and outcome level (e.g., risk of bias), and at review-level (e.g., incomplete retrieval of identified research, reporting bias).	35-36
Conclusions	26	Provide a general interpretation of the results in the context of other evidence, and implications for future research.	37
**FUNDING**			
Funding	27	Describe sources of funding for the systematic review and other support (e.g., supply of data); role of funders for the systematic review.	no funding

Attachment 1

Tab. 2: Reasons for exclusion

Author and Year	Reason for exclusion
Chennoufi et al., 2004	Different intervention
Chan et al., 2018	Phage OMKO1 and ceftazidime
Bruttin et al., 2005	Healthy volunteers
Bachrach et al., 2002	Healthy volunteers
Jikia et al., 2005	PhagoBioDerm
Johnson et al.,	Animal study
Kochetkov et al., 1989	Only abstract in English
Lang et al., 1979	Only abstract in English
Lazareva et al., 2001	Only abstract in English
Leitner et al., 2017	No results published yet
Ligonenko et al., 2015	Only abstract in English
Marei et al., 2003	Different intervention
Markoishvil et al., 2002	PhagoBioDerm
Miedzybrodzki et al., 2008	Analysis of inflammatory markers
Marza et al., 2006	Antibiotic and Phage therapy
Nazari et al., 2011	^{99m}Tc-ubiquicidin 29–41 cationic antimicrobial peptide
Rubinstein et al., 2000	HIV-1 infected patients
Szelachowska et al., 2014	Phage neutralization
Slopek et al., 1985	Method and Intervention unclear
Weber-Dabrowska et al., 1987	Different intervention

Attachment 1

Tab. 3: Summary table of quality assessment of cohort studies (CASP)

Validation of Results								Results			Generalisability			
Author	Focus of Issue	Recruit-ment accept-able	Accurate measure-ment of exposure	Accurate measure-ment of outcome	Identifica-tion of all confound-ing factors	Impact of confounding factors on design/Anal-ysis taken into account	Follow-up of subjects complete	Follow-up long enough	Precise results	Believable results	Applicable to local population	In accor-dance with other available evidence	Implica-tion for practice	Is the study quality high enough?
Patel et al., 2019	X	O	X	X	X	O	X	X	X	X	X	X	X	X
Gupta et al., 2019	X	X	X	X	X	O	X	O	X	X	O	X	X	X
Dabrowska et al., 2003	X	X	X	O	O	O	O	X	X	X	X	X	X	X

X = yes
O = no

Tab. 4: Summary table of quality assessment of randomised controlled trials
(CASP)

**Author**	**Focus of issue**	**Random-isation**	**Patients accounted for con-clusion**	**Blinding**	**Homogeneity of groups**	**Homogeneity of treatment apart from intervention**	**Significant treatment effect**	**Precise treatment effect**	**Applicable to local population**	**Clinically important outcomes considered**	**Benefit**	**Acceptable quality?**
Jault et al., 2018	X	X	X	O	O	X	O	X	X	X	X	X
Totté et al., 2017	X	X	X	X	X	X	O	X	X	X	X	X
Wright et al., 2009	X	X	X	X	NS	X	X	X	X	X	X	X
Rhoads et al.,	X	X	X	X	X	X	O	X	X	X	X	X

Attachment to: Özal D, Arndt A, Thomé M. Bacteriophages and related endolysins for reduction of microorganisms in the human body – a systematic review. GMS Hyg Infect Control. 2022;17:Doc01. DOI: 10.3205/dgkh000404

Attachment 1

Tab. 5: Summary table of case studies (Joanna Briggs Critical Appraisal Checklist)

**Author, Year**	Dedrick et al., 2019	Totté et al., 2017	Jennes et al., 2017	Fish et al., 2018	Schooley et., 2017	Fish et al., 2016
1. Were patient's demographic characteristics clearly described?	Unclear	Yes	Yes	Yes	Yes	Yes
2. Was the patient's history clearly described and presented as a timeline?	Yes	Yes	Yes	Yes	No	Unclear
3. Was the current clinical condition of the patient on presentation clearly described?	Yes	Yes	Yes	Yes	Yes	Yes
4. Were diagnostic tests or assessment methods and the results clearly described?	Yes	No	Yes	Yes	Yes	No
5. Was the intervention(s) or treatment procedure(s) clearly described?	Yes	Unclear	Yes	Yes	Yes	Yes
6. Was the post-intervention clinical condition clearly described?	No	Unclear	N/A	Yes	Yes	Yes
7. Were adverse events (harms) or unanticipated events identified and described?	N/A	Unclear	Yes	Unclear	No	N/A
8. Does the case study provide takeaway lessons?	Yes	Yes	Yes	Yes	Yes	Yes
9. Are the conclusions drawn justified by the results?	Yes	Yes	Yes	Yes	Yes	Yes
10. Are the findings of the study transferable to other settings?	Yes	Yes	Yes	Yes	Yes	Yes

Attachment 1

Tab. 6: Search in Medline and Embase

Search	Search Terms
#1	enzyme therap*.mp. [mp=title, abstract, original title, name of substance word, subject heading word, floating sub-heading word, keyword heading word, organism supplementary concept word, protocol supplementary concept word, rare disease supplementary concept word, unique identifier, synonyms],
#2	bacteriophage*.mp. [mp=title, abstract, original title, name of substance word, subject heading word, floating sub-heading word, keyword heading word, organism supplementary concept word, protocol supplementary concept word, rare disease supplementary concept word, unique identifier, synonyms],
#3	intravenous phage therap*.mp. [mp=title, abstract, original title, name of substance word, subject heading word, floating sub-heading word, keyword heading word, organism supplementary concept word, protocol supplementary concept word, rare disease supplementary concept word, unique identifier, synonyms],
#4	phage treatment*.mp. [mp=title, abstract, original title, name of substance word, subject heading word, floating sub-heading word, keyword heading word, organism supplementary concept word, protocol supplementary concept word, rare disease supplementary concept word, unique identifier, synonyms],
#5	antimicrobial peptide*.mp. [mp=title, abstract, original title, name of substance word, subject heading word, floating sub-heading word, keyword heading word, organism supplementary concept word, protocol supplementary concept word, rare disease supplementary concept word, unique identifier, synonyms],
#6	biopharmaceutic*.mp. [mp=title, abstract, original title, name of substance word, subject heading word, floating sub-heading word, keyword heading word, organism supplementary concept word, protocol supplementary concept word, rare disease supplementary concept word, unique identifier, synonyms],
#7	enzybiotic*.mp. [mp=title, abstract, original title, name of substance word, subject heading word, floating sub-heading word, keyword heading word, organism supplementary concept word, protocol supplementary concept word, rare disease supplementary concept word, unique identifier, synonyms],
#8	endolysin*.mp. [mp=title, abstract, original title, name of substance word, subject heading word, floating sub-heading word, keyword heading word, organism supplementary concept word, protocol supplementary concept word, rare disease supplementary concept word, unique identifier, synonyms],
#9	lysin*.mp. [mp=title, abstract, original title, name of substance word, subject heading word, floating sub-heading word, keyword heading word, organism supplementary concept word, protocol supplementary concept word, rare disease supplementary concept word, unique identifier, synonyms],
#10	artilysin*.mp. [mp=title, abstract, original title, name of substance word, subject heading word, floating sub-heading word, keyword heading word, organism supplementary concept word, protocol supplementary concept word, rare disease supplementary concept word, unique identifier, synonyms],
#11	engineered endolysin*.mp. [mp=title, abstract, original title, name of substance word, subject heading word, floating sub-heading word, keyword heading word, organism supplementary concept word, protocol supplementary concept word, rare disease supplementary concept word, unique identifier, synonyms],
#12	protein engineering.mp. [mp=title, abstract, original title, name of substance word, subject heading word, floating sub-heading word, keyword heading word, organism supplementary concept word, protocol supplementary concept word, rare disease supplementary concept word, unique identifier, synonyms],
#13	engineered phage*.mp. [mp=title, abstract, original title, name of substance word, subject heading word, floating sub-heading word, keyword heading word, organism supplementary concept word, protocol supplementary concept word, rare disease supplementary concept word, unique identifier, synonyms],
#14	viral protein*.mp. [mp=title, abstract, original title, name of substance word, subject heading word, floating sub-heading word, keyword heading word, organism supplementary concept word, protocol supplementary concept word, rare disease supplementary concept word, unique identifier, synonyms],
#15	phage therap*.mp. [mp=title, abstract, original title, name of substance word, subject heading word, floating sub-heading word, keyword heading word, organism supplementary concept word, protocol supplementary concept word, rare disease supplementary concept word, unique identifier, synonyms],
#16	#1 OR #2 OR #3 OR #4 OR #5 OR #6 OR #7 OR #8 OR #9 #10 OR #11 OR #12 OR #13 OR #14 OR #15
#17	antibact*.mp. [mp=title, abstract, original title, name of substance word, subject heading word, floating sub-heading word, keyword heading word, organism supplementary concept word, protocol supplementary concept word, rare disease supplementary concept word, unique identifier, synonyms],

Attachment 1

Search	Search Terms
#18	antibiotic*.mp. [mp=title, abstract, original title, name of substance word, subject heading word, floating sub-heading word, keyword heading word, organism supplementary concept word, protocol supplementary concept word, rare disease supplementary concept word, unique identifier, synonyms],
#19	antimicrobial*.mp. [mp=title, abstract, original title, name of substance word, subject heading word, floating sub-heading word, keyword heading word, organism supplementary concept word, protocol supplementary concept word, rare disease supplementary concept word, unique identifier, synonyms],
#20	antibiotic resistance*.mp. [mp=title, abstract, original title, name of substance word, subject heading word, floating sub-heading word, keyword heading word, organism supplementary concept word, protocol supplementary concept word, rare disease supplementary concept word, unique identifier, synonyms],
#21	bacterial infect*.mp. [mp=title, abstract, original title, name of substance word, subject heading word, floating sub-heading word, keyword heading word, organism supplementary concept word, protocol supplementary concept word, rare disease supplementary concept word, unique identifier, synonyms],
#22	multidrug resistance*.mp. [mp=title, abstract, original title, name of substance word, subject heading word, floating sub-heading word, keyword heading word, organism supplementary concept word, protocol supplementary concept word, rare disease supplementary concept word, unique identifier, synonyms],
#23	drug resistance*.mp. [mp=title, abstract, original title, name of substance word, subject heading word, floating sub-heading word, keyword heading word, organism supplementary concept word, protocol supplementary concept word, rare disease supplementary concept word, unique identifier, synonyms],
#24	anti-bacterial therap*.mp. [mp=title, abstract, original title, name of substance word, subject heading word, floating sub-heading word, keyword heading word, organism supplementary concept word, protocol supplementary concept word, rare disease supplementary concept word, unique identifier, synonyms],
#25	anti-bacterial treatment*.mp. [mp=title, abstract, original title, name of substance word, subject heading word, floating sub-heading word, keyword heading word, organism supplementary concept word, protocol supplementary concept word, rare disease supplementary concept word, unique identifier, synonyms],
#26	antibacterial therap*.mp. [mp=title, abstract, original title, name of substance word, subject heading word, floating sub-heading word, keyword heading word, organism supplementary concept word, protocol supplementary concept word, rare disease supplementary concept word, unique identifier, synonyms],
#27	antibacterial treatment*.mp. [mp=title, abstract, original title, name of substance word, subject heading word, floating sub-heading word, keyword heading word, organism supplementary concept word, protocol supplementary concept word, rare disease supplementary concept word, unique identifier, synonyms],
#28	intravenous antibiotic*.mp. [mp=title, abstract, original title, name of substance word, subject heading word, floating sub-heading word, keyword heading word, organism supplementary concept word, protocol supplementary concept word, rare disease supplementary concept word, unique identifier, synonyms
#29	#17 OR #18 OR #19 OR #20 OR #21 OR #22 OR #23 OR #24 OR #25 OR #26 OR #27 OR #28
#30	human*.mp. [mp=title, abstract, original title, name of substance word, subject heading word, floating sub-heading word, keyword heading word, organism supplementary concept word, protocol supplementary concept word, rare disease supplementary concept word, unique identifier, synonyms],
#31	wound healing*.mp. [mp=title, abstract, original title, name of substance word, subject heading word, floating sub-heading word, keyword heading word, organism supplementary concept word, protocol supplementary concept word, rare disease supplementary concept word, unique identifier, synonyms],
#32	reduction of infec*.mp. [mp=title, abstract, original title, name of substance word, subject heading word, floating sub-heading word, keyword heading word, organism supplementary concept word, protocol supplementary concept word, rare disease supplementary concept word, unique identifier, synonyms],
#33	#31 OR #32
#34	#33 AND #29 AND #16 AND #30
	**EMBASE: 540** **MEDLINE: 281**
